# Environmental Impact Assessment by Green Processes

**DOI:** 10.3390/ijerph192315575

**Published:** 2022-11-24

**Authors:** Aristide Giuliano, Massimiliano Errico, Hamid Salehi, Pasquale Avino

**Affiliations:** 1 ENEA–Italian Agency for New Technologies, Energy and Sustainable Economic Development, Department of Energetic Technologies, Trisaia Research Centre, I-75026 Rotondella, Italy; 2 Department of Green Technology, University of Southern Denmark, Campusvej 55, 5230 Odense, Denmark; 3 Wolfson Centre for Bulk Solids Handling Technology, Faculty of Engineering & Science, University of Greenwich, London SE10 9LS, UK; 4 Department of Agricultural, Environmental and Food Sciences (DiAAA), University of Molise, Via de Sanctis, 86100 Campobasso, Italy

Global primary energy consumption has been steadily increasing since the Industrial Revolution, and it is showing no sign of slowing down in the coming years. This trend is accompanied by increasing concentrations of pollutants in the Earth’s biosystems and general concerns regarding the health and environmental impacts that will ensue. Air quality, water purity, atmospheric CO_2_ concentration, etc., are some examples of environmental parameters that are degrading due to human activities. Pollutant abatement systems, biobased processes, novel environmental assessment tools, and pollutant monitoring equipment and methods can help us to reach environmental neutrality of human activities.

In this background, the Special Issue of the *International Journal of Environmental Research and Public Health* on the Environmental Impact Assessment by Green Processes offers insights into the sustainable conversion processes and environmental assessment using novel monitoring methods and tools useful for policymakers. A significant collection of contributions and studies is presented. Overall, 15 manuscripts were published in this Special Issue after evaluation by the Guest Editors and by many experts involved in the peer-review process.

The collected articles cover a wide range of macro-themes:

- The environmentally friendly conversion processes;
- Novel monitoring systems and technologies;
- Environmental assessment tools and qualifying parameters that are useful for political decision-makers.

De Bari et al. [1] individuated wastewater treatment as a high-environmental-impact process in biorefinery systems, while Giuliano et al. [2] showed that direct DME synthesis can be used as an environmentally friendly strategy for carbon dioxide recycling. Two papers collected in the first group, “the environmentally friendly conversion processes”, studied wastewater from two different points of view. Catizzone et al. [3] developed innovative processes for the purification of wastewater from biomass conversion thermochemical processes. Two commercial activated carbons and two residual biochars obtained through pyrolysis and gasification processes were assessed as potential adsorbents for the purification of wastewater produced in a syngas wet scrubber unit of a biomass gasification plant. Phenol solution was used as a model solution for the investigations. The results indicated the superiority of activated carbons due to the higher pore volume, including biomass-derived char. The phenol adsorption capacity increases from about 65 m g^−1^ for gasification biochar to about 270 mg g^−1^ for commercial activated carbon. Camilleri-Rumbau et al. [4] studied the experimental setup of membrane-based manure and digestate purification processes in a review. The effects of the feed characteristics, membrane operating conditions (pressure, cross-flow velocity, temperature), pH, flocculation–coagulation and membrane cleaning on fouling and membrane performance were presented. Membrane fouling represents one of the major drawbacks when using membrane technologies during farm effluent processing. However, by understanding the related mechanisms and fouling composition and establishing efficient membrane pretreatment and cleaning strategies, membrane technologies can lead to outstanding performance in terms of volume reduction and nutrient recovery.

Four papers were collected in the second group concerning novel monitoring technologies. Lotrecchiano et al. [5] aimed to analyze the air quality data of the monitoring network of the regional agency for environmental protection of the Campania region (Italy), integrated with an innovative monitoring station based on IoT technology to highlight criticalities in the levels of pollution. In-depth analyses showed that two events related to Saharan dust occurred, which led to an increase in the measured PM10 values. These Saharan phenomena were the cause of very high values for PM_10_ (until 260 µg m^−3^). From air quality to the toxicity of heavy metals in an aquatic ecosystem, Adnan et al. [6] presented the acute and chronic toxicity based on luminescence inhibition assay using newly isolated *Photobacterium* sp. NAA-MIE as the indicator. Two different mathematical approaches, Toxicity Unit (TU) and Mixture Toxicity Index (MTI), were used to describe it. The toxicity result of this strain may be effective for taking the toxicity levels of a mixture of pollutants that occur together in ecosystems into theoretical consideration and in the same way contribute extrapolation studies under acute to chronic exposures for mixture metal toxicity in deriving safe limits and standards aimed at protecting organisms in the environment. Sui and Lv [7] applied the environmental Kuznets curve hypothesis and a decoupling analysis to examine the relationship between crop production and agricultural carbon emissions during 2000–2018, and it further provided a decomposition analysis of the changes in agricultural carbon emissions using the log mean Divisia index (LMDI) method. Overall, agricultural economic growth played a significant role in the increase in agricultural carbon emissions, while agricultural carbon emission intensity was the main factor behind the decline in agricultural carbon emissions in China, especially in the years 2003 and 2008, when turning points toward a downward trend in agricultural carbon emissions and strong decoupling states appeared. Different from strong decoupling, both agricultural carbon emission intensity and agricultural labor forces acted as positive driving factors of agricultural carbon emissions, while the agricultural structure and agricultural economic growth acted as inhibitory driving factors of agricultural carbon emissions. Celades et al.’s [8] sampling methodology and mathematical data treatment were developed, which enable us to determine not only total suspended particulates emitted at channeled sources but also the PM_10_, PM_2.5_, and PM_1_ mass fractions and emission factors, using a seven-stage cascade impactor. The proposed methodology was applied to different stages of the ceramic process, including ambient temperature (milling, shaping, glazing) and medium–high-temperature (spray-drying, drying, firing, and frit melting) stages. In total, more than 100 measurements were performed (pilot scale and industrial scale), which leads to a measurement time of 1500 h.

Regarding the environmental assessment tools and qualifying parameters useful for political decision-makers, nine papers were submitted to the Special Issue. In particular, Lin et al. [9] introduced the concept of innovative human capital by developing a new index that measures human capital based on the number of patents for every one million R&D full-time equivalent staff. On the other hand, Wang et al. [10] studied the impact of the Emissions Trading System (ETS) on the Green Total Factor Productivity (GTFP) based on the spatial difference-in-differences approach. The paper resulted in the ETS significantly improving the GTFP of the pilot cities, producing a spatial spillover effect. The results were robust to the placebo test; further analysis showed that the policy effect was mainly driven by improving energy efficiency, promoting green innovation, and optimizing the industrial structure. The third environmental parameter was proposed by Liu et al. [11], using the carbon tax as the main policy tool to promote low-carbon economic development. This had a positive impact on reducing corporate carbon dioxide emissions, promoting the development of more energy-saving emission reduction technologies, and exploring more renewable resources. The research also showed that the levy of a carbon tax will adversely affect economic development, residents’ income, and social welfare. However, introducing a suitable carbon tax recycling mechanism when formulating carbon tax policies can reduce the impact of the carbon tax on related industries. The same research group, Liu et al. [12], reconsidered the carbon tax recovery policy supplemented by technological progress in the clean power sector, showing that it can promote economic growth, improve social welfare, and reduce the intensity of carbon dioxide emissions. The introduction of clean power technology advances makes up for the negative impact of economic growth and social welfare losses, promoting the sustainable growth of the green economy. Advances in clean power technology promote the transformation of the power structure. The advancement of clean power technology drives the production of the clean power sector and replaces thermal power generation. Mehmood Ali Shah et al. [13], differently to previous studies, adopted a mediation model and unfolded not only the role of green human resource practices in the psychological climate and green organizational culture but also clarified the mediating role of the green psychological climate and green organizational culture in sustainable environmental efficiency. The work recommended that a green psychological climate and green organizational culture can be strengthened to achieve more environmental benefits of green human resource management practices in organizations. The results urged researchers to reconsider green human resource management policies for more clarification on the present needs of organizations for developing a green environment. Finally, four works focused on manufacturing activities’ environmental compliance. Liu et al. [14] used the random-effects Tobit model and the double hurdle model to empirically conduct a robustness test. The robust conclusion was that environmental compliance had a significant U-shaped relationship with enterprise innovation, which means that environmental compliance will inhibit enterprise innovation on the left of the inflection point of environmental compliance, while environmental compliance on the right of the inflection point will promote enterprise innovation. How CEO tournament incentives induce top executives to invest more in green innovation was studied by Ullah et al. [15]. The main results supported tournament theory, which proposed that better incentives induced top executives’ efforts to win the tournament incentives, and such efforts were subject to fiercer competition among employees, which improved firms’ social and financial performance. Sunday Adebayo et al. [16] re-assessed the environmental Kuznets curve, taking into consideration the role of hydroelectricity consumption and urbanization. As a result, regulations that decrease the usage of hydroelectricity will hurt economic growth. Any hydroelectricity deficit will also hinder economic progress. Furthermore, a decrease in output will hurt the hydroelectricity demand. Any shock to one of the series of interests will be felt in the others, and the feedback flow will keep the chain going. As a result, expansionary hydroelectricity plans would bring benefits. Hatice Sezgin et al. [17] focused on human development in reducing CO_2_ emissions in the G7 and/or BRICS (Brazil–Russia–India–China–South Africa) economies. According to the G8 countries’ joint declarations, efforts were consistently taken to significantly reduce greenhouse gas emissions and to support sustainable energy and human development. However, as most scholars indicate, there was still a gap between political decisions, on the one hand, and the position of different researchers and analysts regarding the need to further and intensify environmental stringency policies, on the other hand. Our findings indicated that environmental stringency policies and human development were important for environmental sustainability. However, environmental stringency policies can negatively affect economic growth and employment by raising costs at the beginning. However, the countries offset the negative economic effects of environmental stringency policies through innovation, considering the Porter hypothesis and empirical findings over time. On the other hand, improvements in human development were also effective for environmental sustainability.
